# Formation versus Hydrolysis of the Peptide Bond from a Quantum-mechanical Viewpoint: The Role of Mineral Surfaces and Implications for the Origin of Life

**DOI:** 10.3390/ijms10030746

**Published:** 2009-02-26

**Authors:** Albert Rimola, Piero Ugliengo, Mariona Sodupe

**Affiliations:** 1 Dipartimento di Chimica IFM, NIS Centre of Excellence and INSTM (Materials Science and Technology) National Consortium, University of Torino, Via P. Giuria 7, 10125, Torino, Italy; 2 Departament de Química, Universitat Autònoma de Barcelona, 08193, Bellaterra, Spain

**Keywords:** Peptide bond formation, peptide hydrolysis, catalysis, mineral surfaces, theoretical calculations, prebiotic chemistry

## Abstract

The condensation (polymerization by water elimination) of molecular building blocks to yield the first active biopolymers (*e.g.* of amino acids to form peptides) during primitive Earth is an intriguing question that nowadays still remains open since these processes are thermodynamically disfavoured in highly dilute water solutions. In the present contribution, formation and hydrolysis of glycine oligopeptides occurring on a cluster model of sanidine feldspar (001) surface have been simulated by quantum mechanical methods. Results indicate that the catalytic interplay between Lewis and Brønsted sites both present at the sanidine surface, in cooperation with the London forces acting between the biomolecules and the inorganic surface, plays a crucial role to: i) favour the condensation of glycine to yield oligopeptides as reaction products; ii) inhibit the hydrolysis of the newly formed oligopeptides. Both facts suggest that mineral surfaces may have helped in catalyzing, stabilizing and protecting from hydration the oligopeptides formed in the prebiotic era.

## 1. Introduction

The pioneering experiment of Miller [1] evidenced that the first molecular building blocks could have been formed through standard chemical reactions and established a landmark in the field of prebiotic chemistry. More recently, R. M. Hazen [2] suggested that at least a sequence of four steps of increasing chemical complexity are required for the origin of life on Earth to occur: i) the emergence of biomolecules; ii) the emergence of macromolecules; iii) the emergence of self-replicating systems; iv) the emergence of molecular evolution by natural selection. The prebiotic soup theory (*i.e.* Miller-based experiments) only touches the first step: by applying electric discharges to a heated mixture of gases, either resembling highly reducing [1,3–6] (namely H_2_O, NH_3_, CH_4_ and H_2_) or neutral [7–9] (namely H_2_O, N_2_ and CO_2_) primitive Earth atmospheres, the formation of biochemically significant compounds such as amino acids is indeed possible. Similar to processes occurring on the pristine Earth, chemical processes occurring in the interstellar and nebular environments may also lead to the synthesis of biochemical monomers [10–12]. Although the detection of glycine in these media is controversial, [13,14] the presence of a wide variety of amino acids in carbonaceous meteorites fallen on Earth strengthens this theory [15,16]. At the bottom of the Oceanic crust [17], energy sources like hydrothermal vents known as “black-smokers” allowed for carbon fixation [18,19] and formation of the first biomonomers thanks to the reducing power of sulphide minerals coupled to conditions of high temperatures and pressures readily available at those deep-sea sites. Therefore, there are a variety of possibilities for the first step of the life’s emergence sequence to occur. However, more problematic is to find convincing clues as far as the second step is concerned; that is, the emergence of first active biopolymers during primitive Earth from the available building blocks. Providing relevant clues to this point is not trivial, since the formation of biopolymers envisages condensation reactions (*e.g.* amino acids to form peptides) in which water molecules are released. Obviously these processes are disfavoured in the presence of excess water, *i.e.* the hydrolysis of the biopolymers is the thermodynamically favoured process. For instance, focusing on dipeptide formation, while theoretical calculations suggest that the condensation of two alanines in gas-phase is an isoergonic process under normal conditions [20], experimental measures in water solution at neutral pH estimate Δ*G*^0^_310_≈ 4 kcal·mol^−1^ [21]. British biophysicist J. D. Bernal advocated the special role of clay minerals as promoters for the prebiotic polymerization of monomer building blocks [22]. Indeed, clays contain proper adsorption sites that, on one hand, may immobilize and concentrate monomers and protect biopolymers from hydration and, on the other hand, may induce a lowering of the activation barrier because of the presence of catalytic active sites at the surface and in the interlayer regions. Several experiments and works have validated the Bernal’s speculations: i) adsorption and polymerization of amino acids on oxide mineral surfaces [23]; ii) polymerization of nucleotides in the presence of clays [24–26]; iii) peptide formation by activation of amino acids in the presence of sulphide minerals [27,28].

In spite of these experimental evidences, very little is known about the mechanistic steps through which these processes occur. This can be more readily achieved by means of quantum chemical methods by studying possible reaction mechanisms and characterizing the reactive potential energy surfaces (PES). An intriguing example is provided by the work of D. Marx and co-workers, who adopting a metadynamics technique to model the peptide synthesis via glycine adsorption on pyrite surfaces, showed how the extreme temperature-pressure thermodynamic conditions present at hydrothermal vents are crucial for the activation of the process [29–33]. On the other hand, theoretical studies revealed that pure silicates are inert toward peptide bond formation [34], whereas in contrast alumina-silicates activate the process by a certain degree [20,35]. In particular, in a recent work we have demonstrated that the co-presence of Lewis and Brønsted acidic sites at the surface of a feldspar significantly lowers the energy barriers for the condensation of amino acids and the present work, along the same lines, expands on some crucial points not taken into account previously [20]. In particular, remaining questions are: i) since the reaction product (a peptide) remains firmly attached to the surface, will it undergo further condensation reactions with other incoming amino acids to be further elongated?; ii) since water tends to break down biopolymers, will the surface protect the formed peptide towards hydrolysis? In the present work, the same quantum mechanical approach adopted previously was used on a larger cluster mimicking the sanidine feldspar surface where the condensation reactions between three glycine molecules were studied. Additionally, we have also modelled the hydrolysis of the glycylglycine peptide attached to the feldspar surface in the presence of explicit water molecules. Although this study can provide interesting insights on the mechanism of amino acid polymerization on a highly abundant mineral surface, it is worth noting that present calculations simulate just one of the many possible situations. Moreover, due to size and complexity of the system our model and calculations present some limitations and thus, results could be considered quite speculative at some points. First, the adoption of sanidine is somehow arbitrary despite its abundance in the Earth crust, as other feldspars may also be relevant. Second, the substitution of the cations at the surface by protons, needed to arrive at a surface with some chemical reactivity has been assumed to occur in such a way that nearby Brønsted/Lewis sites are present. In real life this process depends on the nature and physico-chemical conditions of the sanidine surrounding and it is usually difficult to assess their location experimentally without large uncertainty. Third, the role of solvation by the ubiquitous presence of water is here modelled in a rather naive way, in line with the available computational resources which do not allow exploiting macroscopic solvated systems within a proper quantum mechanical molecular dynamics simulation. In essence, this work will serve to highlight some key facts in order to stimulate more profound researches not only by means of modelling techniques but, hopefully, by planning well focused experiments.

## 2. Methods

### 2.1. Surface Model

**Figure 1.**
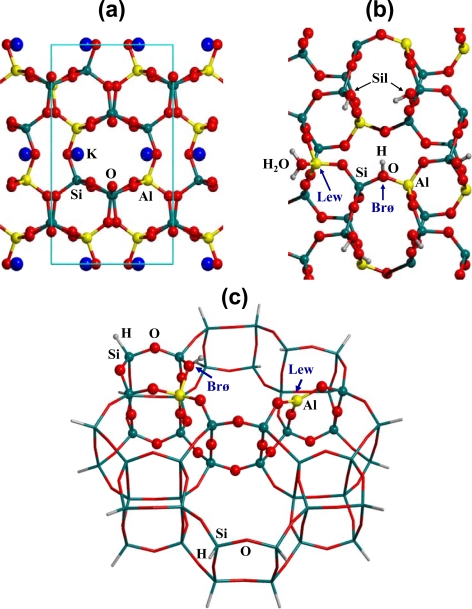
(a) Top view of the bare K-containing sanidine feldspar surface. (b) Top view of the bare H-exchanged sanidine feldspar surface. Lew (Lewis), Brø (Brønsted), and Sil (silanol) active sites are shown. The structure of section a) has been rotated by 90° for clarity of representation. (c) Finite cluster model derived from the H-exchanged sanidine feldspar structure with the co-presence of Lewis (Lew) and Brønsted (Brø) sites. Atoms shown as balls belong to the ONIOM2 high-level B3LYP zone; real zone (shown as sticks) is treated at the MNDO level.

Following a suggestion by Smith [36], peptide bond formation and its hydrolysis have been studied in the presence of the sanidine feldspar mineral, an aluminosilicate that covers approximately the 60% of the Earth’s crust. Feldspars envisage a silica-based framework in which Ca^2+^, Na^+^ or K^+^ are present as charge-balancing cations for the Al substitution (see Figure 1a). The weathering of feldspar surfaces, together with fluctuating thermal conditions, allow for the exchange of some of the cations by protons, resulting in surfaces rich in Lewis and Brønsted sites, as happens for the preparation of acidic zeolites used as cracking catalysts. Figure 1b shows a plausible model for a H-exchanged sanidine (001) surface, in which terminal silanols (Si-OH), acidic Brønsted sites and Lewis sites (with a coordinated water) are all present as active surface sites. It is worth recalling that the (001) surface is the one naturally occurring for sanidine feldspar. A large and realistic finite molecular cluster has been extracted from the H-exchanged sanidine feldspar surface model which includes the most important catalytic sites (see Figure 1c). Note that Lewis and Brønsted sites are in close spatial proximity, thus allowing a synergic action with respect to both amino acids adsorption and peptide bond formation catalysis.

### 2.2. Computational Details

The structure of the reactants, transition states and products were optimized using the ONIOM2 method [37], combining the hybrid B3LYP [38,39] functional with the 6–31+G(d,p) basis set for the high-level zone and the modified neglect of differential overlap (MNDO) [40] Hamiltonian for the low-level real system. To improve the accuracy of the ONIOM2 energies, once the stationary points were located, the energy was re-evaluated by performing single-point energy calculations at the full B3LYP/6–31+G(d,p) level on the optimized ONIOM2 geometries. For consistency, glycine molecules are always included in the high-level zone and thus, treated at the B3LYP/6–31+G(d,p) level. This combination was proved to be a good compromise between accuracy and computational cost for modelling silica-based materials [41,42], including reactivity [34].

All calculations were performed using the Gaussian03 program [43]. Structures have been characterized by the analytical calculation of the harmonic frequencies as minima (reactants and products) and saddle points (transition states). The Gibbs free energies for all considered structures have been computed by adding the ONIOM2 enthalpy and entropy contributions derived from the standard rigid rotor/harmonic oscillator formulas [44] onto the B3LYP/6–31+G(d,p)//ONIOM2 electronic energies. As observed in a previous work [20], dispersive forces are expected to be relevant for the amino acids’ condensation energy profiles. Because the adopted methodology does not take into account the dispersion originated by the fluctuating instantaneous dipoles, a rather simple strategy is adopted, to at least partially take them into account. In particular, the DFT+D method recently proposed by Grimme [45], which has been proved to be very effective for a number of cases where dispersive interactions are relevant, is adopted. The dispersive correction to the B3LYP/6–31+G(d,p)//ONIOM2 energies is computed, in a *posteriori* fashion, by using the Grimme routine as encoded in the MOLDRAW program [46], to compute the D term and adding the resulting contribution to the B3LYP Δ*G*_298_ to give the final Δ*G*_298_+D total energies. In this way the dispersive contributions are added in a non-self consistent way to the free energies which may somehow underestimate their relevance.

## 3. Results and Discussion

### 3.1. Amino acid polymerization

The condensation between two amino acids envisages a nucleophilic attack of the **N-**amino of one amino acid toward the COOH group of the other amino acid, followed by the release of one H_2_O molecule, thus giving rise to the formation of the *HN-C=O* peptide bond. The energetics for this process in the gas-phase (computed at the B3LYP/6–31+G(d,p) level) at normal conditions exhibit a rather high kinetic free energy barrier, by about 50 kcal mol^−1^, and a reaction free energy around −1 kcal·mol^−1^ [20]. In this section theoretical results addressed to understand the role of the sanidine feldspar surface (F) on the condensation of two and three glcyine (G) molecules to form glycylglcyine (GG) and glycylglycylglcyine (GGG) oligopeptides, respectively, are reported.

For all considered reactions, it has been assumed that G is in its neutral state, this latter being essential for the nucleophilic reactions needed for the condensation process to occur. This is in contrast with the zwitterionic nature of G in presence of an excess of water so that it must be assumed that a fraction of zwitterionic G should convert (at an extra energetic cost) to the neutral form before reacting. An alternative path would be to assume that condensation reactions become possible during the drying cycle of a wetting/drying cycle which readily occurs under natural conditions.

**Figure 2.**
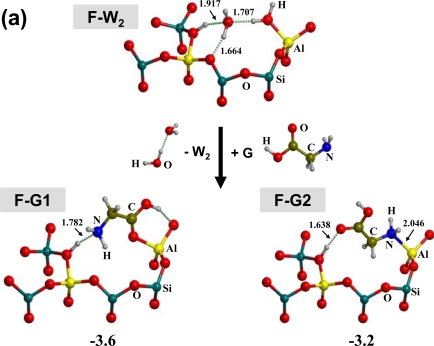

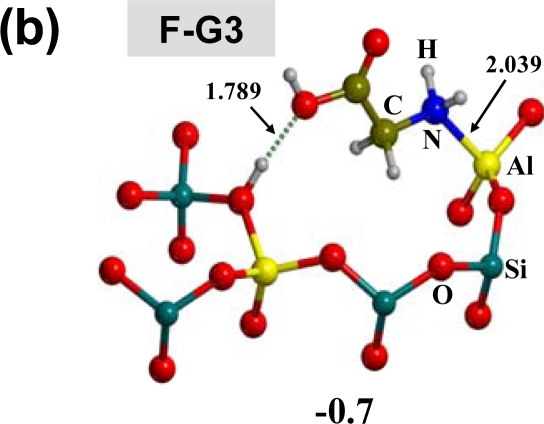
(a) ONIOM2-optimized geometries of the sanidine feldspar structure in interaction with either two H_2_O molecules (**F-W2**) or one glycine molecule (**F-G1** and **F-G2**). Reaction free energies, Δ*G*_298_+D (kcal mol^−1^), for the water displacement by glycine as bare numbers. (b) ONIOM2-optimized geometry of the sanidine feldspar structure in interaction with glycine in a form activated towards nucleophilic attack by an incoming glycine (see text for further details). Only the high level zone is shown for brevity. Distances in Å.

Nonetheless, previous to the polymerization reaction, it is first interesting to know whether the interaction of G with the surface is favourable or not. Considering that the mineral surface is usually hydrated, we have firstly considered F in its micro-solvation state, *i.e.* in the presence of two water molecules (those most tightly bound to the surface) interacting with the Lewis and Brønsted active sites (**F-W_2_**, see Figure 2a), to at least mimic the water molecules present in the medium that interact with the active sites. Thus, the very first stage is to replace the adsorbed H_2_O molecules by G itself. Different G adsorption modes onto F are possible since the NH_2_ and COOH groups may interact with both the Lewis and Brønsted sites. Figure 2a shows all possible adducts: **F-G1** in which the COOH and the NH_2_ group interact with the Lewis and Brønsted sites, respectively, and **F-G2** in which the opposite occurs. For both cases G adsorbs more strongly than the two H_2_O molecules, as the reaction Δ*G*_298_+D values show (see Figure 2a), both structures being almost equiprobable (**F-G2** only 0.4 kcal mol^−1^ above **F-G1**).

Neither **F-G1** nor **F-G2** are suitable for a condensation reaction with an incoming glycine [20]: rotation of the COOH group of **F-G2** is needed to bring the OH group in direct contact with the acidic Brønsted proton (see **F-G3** in Figure 2b) which is then a suitable arrangement to allow a water molecule to become the leaving group during the condensation reaction. **F-G3** is still favourable with respect to water displacement, the reaction free energy being −0.7 kcal/mol (2.9 kcal mol^−1^ above **F-G1**). Its relative population compared to **F-G1** is ≈ 7×10^−3^, high enough on a geological timescale to provide a sufficient amount of **F-G3** for the condensation reaction.

**Figure 3.**
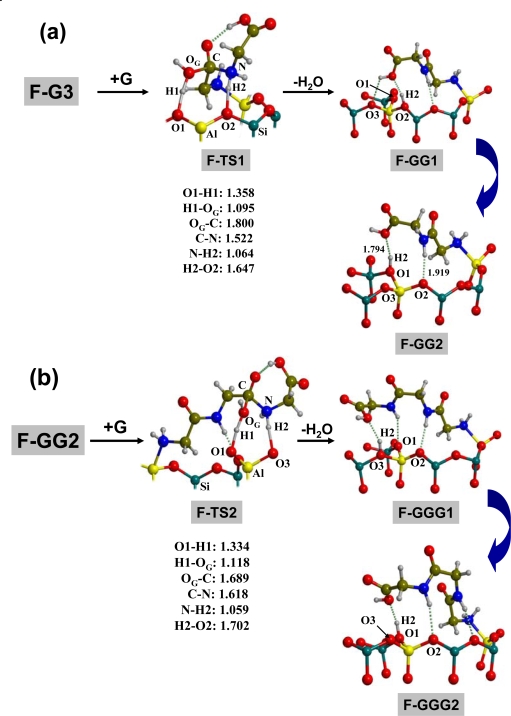
(a) ONIOM2-optimized geometries for the condensation of glycine adsorbed onto sanidine feldspar surface (**F-G3**) with another incoming glycine (**G**) resulting in a dipeptide attached to the surface (**F-GG1** and **F-GG2**). (b) Same as section a) starting from **F-GG2** to give tripeptides attached to the surface (**F-GGG1** and **F-GGG2**). Distances in Å.

Figure 3a shows the peptide bond formation reaction between **F-G3** with the incoming G. This proceeds through an eight-membered ring transition state (**F-TS1**), in which three simultaneous steps take place: i) a proton transfer from the Brønsted site to the glycine OH group (H1**→**O_G_, see Figure 3a) followed by the release of a H_2_O molecule; ii) nucleophilic attack from the amino N atom of the incoming G toward the carboxyl C atom of the adsorbed G (N**→**C, see Figure 3a); iii) a proton transfer from the incoming amino group to a surface oxygen atom (H2 **→**O2, see Figure 3a), displacing the original Brønsted site to a new surface position. The resulting GG product remains adsorbed at the surface, similarly to what happens for **F-G3** *(i.e.* NH_2_ and COOH interacting with the Lewis and Brønsted sites, respectively) including a new H-bond between the peptide NH group and a surface oxygen atom. Interestingly, **F-GG1,** although being the product directly connected to **F-TS1**, is not the most stable product because of the displacement of the original Brønsted site (AlO2-H2, see Figure 3a). Indeed, when the original Brønsted site is regenerated as in **F-GG2** a gain of about 3 kcal mol^−1^ is computed. Proton mobility in acidic zeolites is a well-known phenomenon for which the energy barriers spans the 5–20 kcal mol^−1^ range [47–49], accounting for an easy conversion of **F-GG1** towards the final product **F-GG2**. As mentioned, GG remains attached to the surface and the COOH group is still activated toward further nucleophilic attack due to the interaction with the Brønsted site. This is indeed the case, as Figure 3b shows. The mechanistic steps for this second condensation reaction are very similar to the ones computed for the first condensation; *i.e.* the formation of an eight-membered ring transition state because of the simultaneous proton transfers and the N**→**C attack (**F-TS2**). Additionally, the **F-GGG1** direct product may evolve to **F-GGG2** (1.7 kcal mol^−1^ more stable), in which a surface proton jump transforms the AlO3-H2 into AlO1-H2 Brønsted site (see Figure 3b). Therefore, according to these mechanism paths, the Lewis site only acts as glycine blocker, whereas the Brønsted site activates the condensation of glycine molecules via acidic proton transfer. Let us now analyse the energetics of these processes.

**Figure 4.**
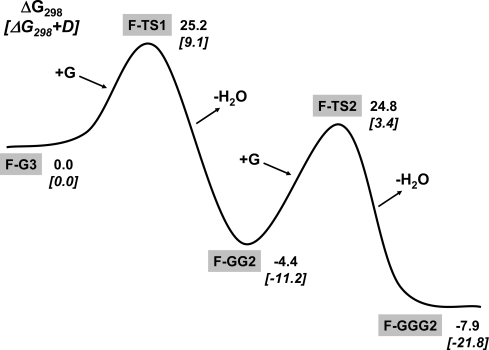
Relative free energy profile (kcal mol^−1^) of the glycine condensation reactions shown in Figure 3. Bare values refer to pure Δ*G*_298_, italic values in brackets refer to Δ*G*_298_+D, including the Grimme’s dispersive correction. Relative free energies refer to the **F-G3** + 2G reference state.

Figure 4 depicts the relative free energy profile of the two condensation reactions assuming **F-G3** + 2G as the reference state. Related to the first condensation, it is worth mentioning that the Δ*G^≠^*_298_ energy barrier is approximately 25 kcal mol^−1^ (half of the gas-phase un-catalyzed process), thus evidencing a relevant activation given by the mineral surface. This significant lowering of the kinetic barrier is due to the formation of a less constrained transition structure (**F-TS1**, eight-membered ring) compared to the four-membered ring formed in the gas-phase. Furthermore, if the dispersive forces are accounted for, the energy barrier (now Δ*G^≠^*_298_+D) becomes as low as 9 kcal mol^−1^, resulting in an even more dramatic free energy barrier lowering.

Even more important, dispersion dramatically contributes in stabilising the F-GG2 product since the computed Δ_r_*G*_298_ and Δ_r_*G*_298_+D reaction free energies are −4 and −11 kcal mol^−1^. Therefore, for the present system, the effect of the dispersive forces occurring between the surface and the bio-moiety are substantial in the stabilization of both transition states and reaction products.

This is also the case for the second condensation, which proceeds through a Δ*G^≠^*_298_+D energy barrier by about 14 kcal mol^−1^ to reach the **F-GGG2** product with a Δ_r_*G*_298_+D energy stabilization of −10.6 kcal mol^−1^ (taking **F-GG2** as reference). It is worth noting that, in essence, the condensation of two amino acids (2G **→** GG + H_2_O) and the condensation of the dipeptide with a third amino acid (GG + G **→** GGG + H_2_O) are equally favoured by a low free energy barrier and a large and negative reaction free energy in the presence of the sanidine feldspar surface. The fact that the two condensation reactions may be considered as two independent processes and owing to their similar energetic requirements suggests that the occurrence of the amino acids polymerization at the surface may be an iterative process. That is, after the first amino acid condensation, the resulting peptide product is likely to be activated for the addition of a further glycine molecule if the surface presents enough Brønsted acidic sites. In this way an easy elongation of the oligopeptide is envisaged, the sanidine surface playing the same leading role at each reactive step. Furthermore, since the reaction free energies are larger and more negative at each step of amino acid condensation (−11.2 and −21.8 for the GG and GGG formation, respectively), it is expected that the newest peptides formed will stay more and more tightly adsorbed at the surface at each elongation step.

### 3.2. Peptide hydrolysis

In the previous section it has been shown that the presence of the sanidine surface favours the peptide bond formation between glycine molecules from both thermodynamic and kinetic points of view. However, in highly diluted water solutions the reverse reaction is known to occur, *i.e.* peptide hydrolysis, so that an intriguing question arises: is hydrolysis also favoured when the oligopeptide is attached to the sanidine feldspar surface? To study this point, we have computed different processes (peptide bond hydrolysis and peptide desorption) by adopting a microsolvation approach (*i.e.* in the presence of a finite number of explicit H_2_O molecules) in order to study the effect of water solvent on the stability of the peptides and to understand the role, if any, of the sanidine feldspar surface.

**Figure 5.**
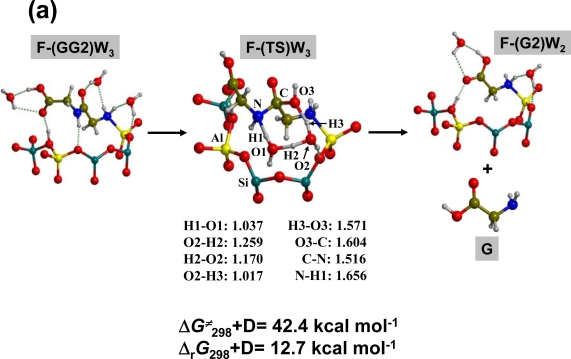

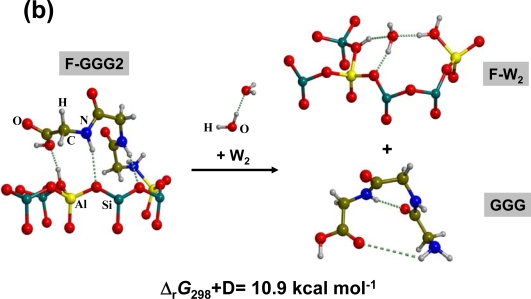
(a) ONIOM2-optimized structures (bond distances in Å) and energetics for the hydrolysis reaction of the GG dipeptide attached to the sanidine feldspar surface in the presence of three H_2_O molecules. **F-(GG2)W_3_** as a reference state. (b) Displacement process of GGG adsorbed at the surface by two H_2_O molecules. **F-GGG2** + W_2_ as the reference state.

For oligopeptides, hydrolysis can occur in different positions, among which two major situations can be distinguished: i) hydrolysis of the COOH terminal peptide bond; ii) hydrolysis of an internal peptide bond. In the first case, the reaction mechanism would just be the reverse of the peptide bond formation shown in the previous section; that is, hydrolysis would occur through the Brønsted site of the surface and, for instance, for **FGGG2** + H_2_O **→ FGG2** + G, Δ*G^≠^*_298_+D and Δ_r_*G*_298_+D would be 25.6 and 10.6 kcal·mol^−1^, respectively (see Figure 4). In the second case, hydrolysis would not be assisted by the Brønsted site but through the water molecules present in the medium. This situation has been modelled for the simplest **F-GG2** system in the presence of three H_2_O molecules and the results are summarized in Figure 5a. The **F-(GG2)W_3_** reactant structure envisages GG adsorbed at the surface while H_2_O molecules maximise the H-bond interactions with the most probable interacting sites in GG (carboxylic, peptide and amino groups). The three H_2_O molecules are all involved in the transition state for the peptide bond hydrolysis by adopting a mechanism which enhances the proton donor/acceptor character of water via the formation of a concerted proton transfer within the eigth-membered ring of **F-(TS)W_3_**. Despite the low-constrained ring formed in **F-(TS)W_3_** the free energy barrier for the peptide hydrolysis is about 42 kcal mol^−1^, with a free reaction energy of about 13 kcal mol^−1^ showing that hydrolysis of the peptide bond is a hindered process for an oligopeptide captured at the feldspar surface. The second relevant process is that in which the adsorbed oligopeptide is desorbed from the surface by the water action. Desorption of glycine by two H_2_O molecules is unfavourable by about 3 kcal mol^−1^ (reverse process of Figure 2a). Figure 5b shows the optimized stationary points involved in the displacement reaction in which GGG attached to the surface is exchanged by two H_2_O molecules. The computed reaction free energy is 10.9 kcal mol^−1^, more endergonic than for the glycine case. These two reaction values suggest that the larger the peptide, the stronger the adhesion. This trend is mainly caused by the dispersive interactions between the surface and the oligopeptide, since the values for the dispersion contributions are 3.5 and 12.5 kcal mol^−1^ for the G and GGG cases, respectively, in favour of the oligopeptide attachment. These values should be taken with some extra caution because the role of water in solvating both the reactants and the products have not been considered. At present, it is outside our computational facilities to model the effect of water molecules on the considered reactions and will be the subject of future studies.

## 4. Concluding Remarks

The formation and hydrolysis of the peptide bond in the presence of a model of sanidine feldspar surface rich in both Lewis and Brønsted sites have been studied quantum mechanically using the ONIOM2[B3LYP/6–31+G(d,p):MNDO] method and including the contribution of dispersive forces in a posterior fashion by means of an empirical London type correction to the quantum total energy.

B3LYP/6–31+G(d,p)//ONIOM2 computed energies show that both the condensation of two and three glycine molecules in the presence of the feldspar surface have kinetic Δ*G^≠^*_298_+D energy barriers as low as 10 kcal mol^−1^ with free reaction Δ_r_*G*_298_+D energies large and negative. The mechanistic steps for these processes suggest that the co-presence of Lewis/Brønsted sites is a key factor in favouring the condensations: the Lewis site firmly binds the glycine to the surface via the NH_2_ group whereas the Brønsted site activates the C=O group towards nucleophilic attack by the incoming glycine. In contrast, the hydrolysis of the peptide bond, here modelled by the attack of three H_2_O molecules toward glycylglycine attached to the surface, has been computed to be an unfavourable process because of the very high Δ*G^≠^*_298_+D barrier and positive reaction Δ_r_*G*_298_+D free energy. Accordingly, in the presence of the sanidine feldspar surface (as a reference system) the peptide bond formation predominates with respect to its hydrolysis. This is indeed in line with the intuition of J. D. Bernal [22], who in the early 50’s suggested the role of mineral surfaces as suitable promoters for biopolymerisation in the prebiotic era.

Finally, it is shown that peptide products can hardly be washed away from the feldspar surface because dispersive interactions and relatively strong H-bond interactions with the surface hinder the desolvation by the water action. These facts are in agreement with the suggestions of L. E. Orgel [50], who stated that: i) the oligomers may be elongated indefinitely by repeated cycles at mineral surfaces; ii) the affinity of a mineral surface for an oligomer increases with the length of the latter, so that the adsorption must become essentially irreversible for sufficiently long oligomers. Our results, although rather speculative, reinforce the idea that inorganic surfaces could have been coated by primitive large polypeptide chains that could have acted as biological templates (that is, exhibiting a pro-enzymatic functionality) for the synthesis of the first biologically relevant macromolecules [51].
